# Long-term survival after surgical aortic valve replacement among patients over 65 years of age

**DOI:** 10.1136/openhrt-2015-000338

**Published:** 2016-03-25

**Authors:** Mansour T A Sharabiani, Francesca Fiorentino, Gianni D Angelini, Nishith N Patel

**Affiliations:** 1 Academic Cardiac Surgery, National Heart & Lung Institute, Imperial College London, London, UK; 2 Bristol Heart Institute, University of Bristol, Bristol, UK

**Keywords:** QUALITY OF CARE AND OUTCOMES

## Abstract

**Objective:**

Surgical aortic valve replacement (AVR) remains the gold standard therapy for severe aortic stenosis. Long-term survival data following AVR is required. Our objective was to provide a detailed contemporary benchmark of long-term survival following AVR among elderly patients (≥65 years) in the UK.

**Methods:**

We conducted a retrospective cohort study of 1815 adult patients undergoing surgical AVR± coronary artery bypass graft (CABG) surgery at a single UK centre between 1996 and 2011. Our main outcome was patient survival, which was assessed by linkage to census records at the Office for National Statistics.

**Results:**

The mean age of the cohort was 75 (±5.6) years. Patients in the AVR alone group had a slightly higher median survival of 10.9 (95% CI 10.5 to 11.8) years than the AVR+CABG group which had a median survival of 9.6 (95% CI 8.7 to 10.1) years (p=0.001 of log-rank test (LRT) for equality of survivor functions). The presence of chronic kidney disease, severely impaired left ventricular function or being a current smoker were each associated with a ≥50% increased risk of long-term mortality. Comparison of our study cohort patients and the reference (operation year, age and gender matched) UK population suggested no difference in survival probability up to 8 years (p=0.55). However, for longer periods of follow-up, the difference became increasingly significant (p<0.0001).

**Conclusions:**

Long-term survival following surgical AVR in patients over 65 years of age is excellent and up to 8 years is comparable to the matched general population.

**Key questions**

**What is already known about this subject?**

- Surgical aortic valve replacement (AVR) in elderly patients has excellent short-term and mid-term outcomes. The establishment of a robust surgical benchmark of long-term survival after open AVR is of increasing importance, particularly in the era of transcatheter aortic valve implantation (TAVI).

**What does this study add?**

- This study provides long-term survival data for patients over 65 years of age undergoing AVR and compares this to the expected survival of the general population. It demonstrates that long-term survival in this patient group is excellent.

**How might this impact on clinical practice?**

- Outcomes following AVR in elderly patients are excellent and, therefore, age should not be a factor when considering patients for surgical AVR. This study provides a benchmark for ongoing trials of TAVI in intermediate-risk patients with severe aortic stenosis.

## Introduction

Aortic valve disease is the most common type of valvular heart disease in Europe and North America, occurring in 2–7% of the population over 65 years of age.^1^ ^2^ When untreated, symptom progression is rapid and lethal, with a median survival of <2 years in those with heart failure symptoms.^3^ ^4^ Medical therapy is largely ineffective for the long-term management of aortic valve disease, and valve replacement remains the standard of care in patients with an acceptable risk profile. Among the oldest patients and those with a high burden of comorbidities, the perioperative risk of surgical aortic valve replacement (AVR) is often perceived to outweigh the potential long-term benefits, with a perception of high operable risk accounting for 30% of all non-operated patients with symptomatic aortic stenosis (AS).^5^ Although the perioperative risk associated with AVR has been well studied, the long-term outcomes after open AVR have not been well described, making accurate assessment of the benefits of open AVR challenging in this population.

Transcatheter delivery of aortic valve prostheses offers a potential treatment option in high-risk patients and has been shown at 2-year follow-up to be superior to medical therapy in non-operable patients and non-inferior to open valve replacement in high-risk patients.^6^ ^7^ As long-term transcatheter AVR outcomes accrue, the establishment of a robust surgical benchmark of long-term survival after open AVR is of increasing importance to inform prognosis and to aid clinical and economic evaluations of new technologies.

The objective of this study was therefore to describe in-hospital outcomes and long-term survival of patients undergoing surgical AVR, to compare their survival with the survival of the age-matched and gender-matched section of the general population and to assess the association between mortality and specific high-risk comorbidities.

## Methods

This was a retrospective cohort study of prospectively collected data from consecutive patients undergoing AVR at the Bristol Heart Institute, Bristol Royal Infirmary, UK, between April 1996 and December 2011. The study was approved by the Clinical Audit Committee of the University Hospitals Bristol National Health Service Foundation Trust to meet ethical and legal requirements, and individual consent was waived. The data collection form is entered in a database (Patient Analysis & Tracking System; Dendrite Clinical Systems, London, UK) and includes five sections that are filled in consecutively by anaesthetists, surgeons, intensive care unit, high dependency unit and ward nurses. Data entry is periodically checked for accuracy by independent database managers.

Chronic kidney disease (CKD) was defined as a baseline serum creatinine of ≥200 μmol/L. Chronic lung disease was defined as chronic obstructive pulmonary disease or asthma requiring bronchodilators or steroid therapy. Diabetes mellitus was defined as patients requiring oral hypoglycaemic agents or subcutaneous insulin therapy. Left ventricular (LV) systolic function was defined as normal (LV ejection fraction (LVEF) ≥52%), mildly impaired (LVEF 41–51%), moderately impaired (LVEF 30–40%) and severely impaired (LVEF <30%) according to the American Society of Echocardiography recommendations.^8^

### Study population

The study cohort is a subset of the Bristol PATS database, which contains 21 515 entries. The study cohort included elderly patients (aged ≥65 years) who had undergone either elective or urgent AVR operation with or without coronary artery bypass graft (CABG) procedure. Records were excluded if they involved multiple valve procedures, other major non-valve-related operations (other than CABG), previous AVR and emergent or salvage operations.

### Patient subgroups

Because long-term outcomes are expected to vary according to the burden of specific comorbidities, several patient subgroups were prospectively identified for analysis. For all analyses, patients were categorised by procedure type (eg, isolated AVR, AVR+CABG) and age (65–69, 70–79, ≥80 years). Other patient subgroups included European System for Cardiac Operative Risk Evaluation (EuroSCORE; low≤5, high≥5) and development of a postoperative complication.

### Study end point

Patient survival, which was the primary outcome of interest for this study, was assessed by linkage to census records at the Office for National Statistics (ONS). Life status (mortality) information for all patients was obtained from the ONS up to 31 July 2013.

### Comparison with the operation year-matched, age-matched and gender-matched UK population

To establish a comparative population-based reference survival curve, we generated a baseline Kaplan-Meier (KM) curve for an age-matched, gender-matched and year-matched patient population using the life tables of ONS.^9^ For each patient in our cohort, we used the annual mortality probabilities from the ONS life tables to run Monte Carlo (MC) simulations for that individual's survival, starting from the year of operation and advancing in yearly increments.^10^ The patient-level events were then aggregated across the cohort to generate a final aggregate KM curve, representing the expected survival behaviour of the matched general population. For each patient, 1000 MC runs were performed to generate statistically sufficient events and produce a reliable baseline survival curve.

### Statistical analysis

Descriptive statistics included mean±SD and percentages to describe patient and operative characteristics in two procedural groups, that is, those who underwent AVR alone and those who underwent AVR+CABG. These procedural groups were compared using t test or χ^2^ test as appropriate. Multiple logistic regression was used to estimate the predictors of in-hospital and 30-day mortality. Median survival times after surgery were measured for various strata of patients. Log-rank test (LRT) was used to evaluate the equality of the survivor functions. Multivariable Cox proportional hazards (CPH) model was used to estimate the HRs of long-term risk predictors of mortality. The assumption of proportionality of hazards was checked visually. For multiple logistic regression and CPH models, all the variables were included initially and stepwise backward elimination approach was used to arrive at the final models. The predictors which were examined for short-term and long-term risk models included age at operation, gender, smoking status, CABG procedure, LV function (LVF), history of chronic lung disease and/or CKD, extracardiac arteriopathy, preoperative arrhythmia, being under hypertensive and/or diabetic treatment as well as the EuroSCORE. We also performed univariate analysis and the p value threshold was Bonferroni adjusted to account for multiple testing.

Statistical analyses including generation of the aggregate KM curve using the simulated MC runs were performed using Stata V.11.2 for Windows (StataCorp, College Station, Texas, USA). MC simulations were performed using R programming language (R Core Team. R: A language and environment for statistical computing. Vienna, Austria: R Foundation for Statistical Computing. 2014).

## Results

### Study population

**Figure 1:**
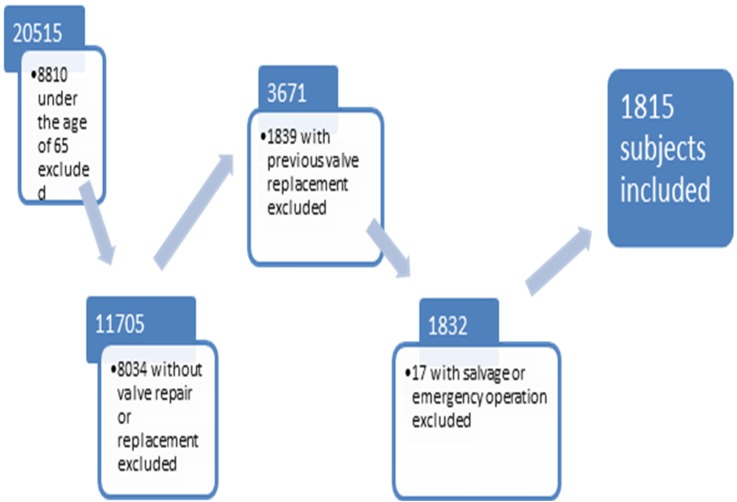
Bristol cardiac surgical database cohort development flow chart.

A cohort of 1815 adult patients underwent AVR for AS between 1996 and 2011, including 967 who underwent isolated AVR and 848 patients who underwent AVR with concomitant CABG surgery (figure 1). They had a mean age of 75 (±5.6) years, 1022 (56%) were male patients, 38 (2.1%) had CKD, 256 (14.1%) had chronic lung disease and 107 (5.9%) had severely impaired LVF. Patient and operative characteristics stratified by procedure are shown in table 1. Thirty (1.7%) patients were lost to long-term follow-up.

**Table 1:** AVR cohort characteristics stratified by procedure (AVR alone, AVR+CABG)

Characteristics	Valve alone	Valve+other	p Value
Number of patients	967	848	
Age at procedure	74.8 (5.6)	75.4 (5.7)	0.02
Male	457 (47.6%)	565 (66.6%)	<0.0001
Cumulative bypass time	92.8 (27.3)	125.6 (38)	<0.0001
Cumulative cross clamp time	67 (18.1)	87.2 (23.1)	<0.0001
EuroSCORE	6.8 (1.9)	7.3 (2.1)	<0.0001
Logistic EuroSCORE	0.079 (0.05)	0.095 (0.075)	<0.0001
High EuroSCORE (>5)	720 (74.5%)	666 (78.5%)	<0.0001
Aortic valve gradient (mm Hg)	55.1 (41.1)	43.8 (37.0)	0.53
Aortic valve (mechanical)	181 (18.7%)	149 (18.2%)	0.11
Severely impaired left ventricular function	49 (5.1%)	58 (6.9%)	0.68
Chronic kidney disease (SCr ≥200 µmol/L)	19 (2.0%)	19 (2.3%)	0.45
Chronic lung disease	142 (14.7%)	114 (13.4%)	<0.0001
Number of grafts	0.0 (0.0%)	1.8 (1%)	=0.21
**Aortic valve size (mm)**			
17	6 (0.6%)	8 (0.9%)	
19	249 (25.8%)	186 (21.9%)	
21	394 (40.87%)	333 (40.1%)	
23	233 (24.1%)	226 (26.6%)	
25	61 (6.3%)	71 (8.4%)	
27	19 (2.0%)	18 (2.1%)	
29	2 (0.2%)	5 (0.6%)	

Mean (SD) or %; t test and χ^2^ tests for comparing means and percentages, respectively.

AVR, aortic valve replacement; CABG, coronary artery bypass graft; EuroSCORE, European System for Cardiac Operative Risk Evaluation.

### In-hospital outcomes

In-hospital outcomes are presented in table 2. A total of 68 (3.8%) patients died in hospital or within 30 days following the operation. Of them, 27 (1.5%) patients were in the AVR group and 41 (2.3%) in the AVR+CABG group. EuroSCORE >5 was a significant predictor of in-hospital/30-day mortality (OR 1.4 (95% CI 1.2 to 1.5), p<0.0001). Postoperative reoperation for bleeding (8.6% vs 7.9%, p=0.048) and cerebrovascular accident (CVA) (1.7% vs 0.7%, p=0.040) was significantly higher in the AVR alone group compared to patients undergoing AVR+CABG. Conversely, the need for postoperative haemofiltration was lower in the AVR group than the AVR+CABG group, although this did not reach statistical significance (1.8% vs 3.2%, p=0.068; table 2). Results of the univariate analysis are shown in online supplementary table S1.

10.1136/openhrt-2015-000338.supp1 Supplementary table Univariate Logistic Regression

**Table 2:** Postoperative complications by procedure type

Postoperative complication	AVR	AVR+CABG	p Value
Number of patients	967	848	
In-hospital death	27 (1.5%)	41 (2.3%)	0.022
Reoperation for bleeding	54 (8.6%)	67 (7.9%)	0.048
Permanent stroke	15 (1.7%)	5 (0.7%)	0.040
Haemofiltration	16 (1.8%)	25 (3.2%)	0.068

AVR, aortic valve replacement; CABG, coronary artery bypass graft.

### Long-term survival after AVR

Survival analysis included 5.4, 17.3 and 10 950.55 years of median, maximum and total time at risk, respectively. In total, 707 (38.9%) patients died during the follow-up period, of which 356 (19.6%) underwent isolated AVR and 351 (19.3%) underwent AVR+CABG. Patients in the AVR alone group had a slightly higher median survival of 10.9 (95% CI 10.5 to 11.8) years than the AVR+CABG group which had a median survival of 9.6 (95% CI 8.7 to 10.1) years (p=0.001 of LRT for equality of survivor functions).

**Figure 2:**
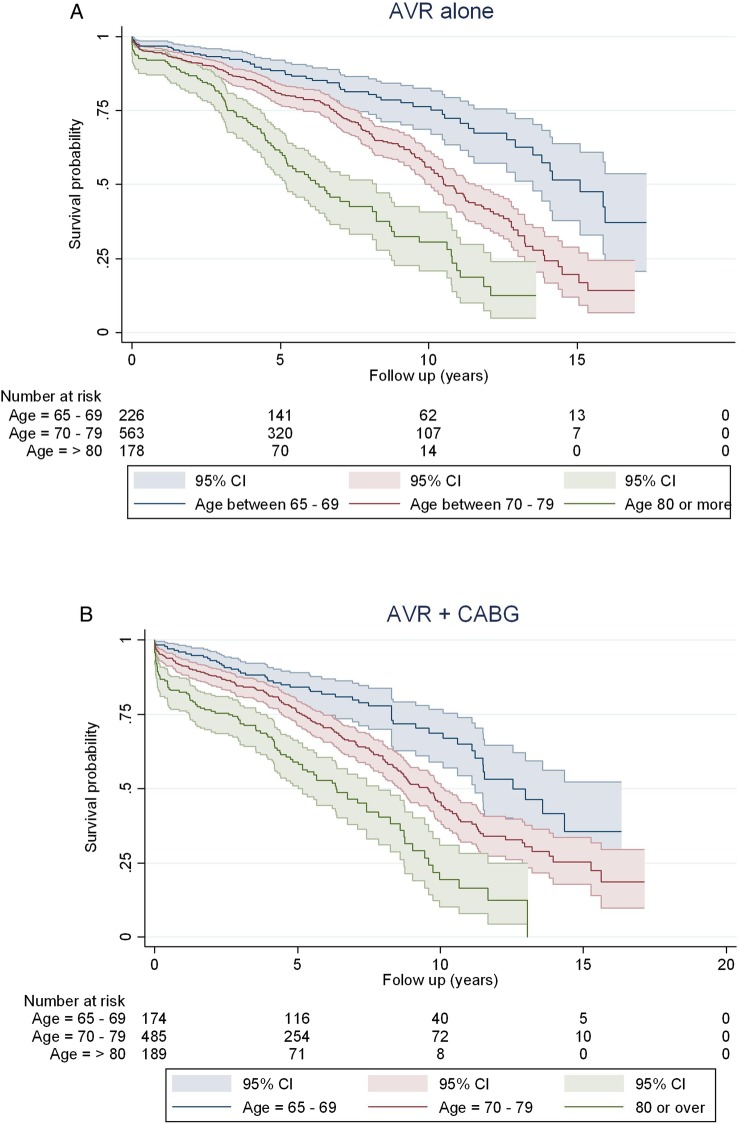
Long-term survival after (A) AVR and (B) AVR+CABG. Survival curves (Kaplan-Meier estimates) are presented with 95% CIs and are stratified by age group. AVR, aortic valve replacement; CABG, coronary artery bypass graft.

Median survival times in the AVR alone group across the age groups of ‘65–69’, ‘70–79’ and ‘80 or over’ were 15.1, 10.6 and 6.3 years, respectively. In the AVR+CABG group, median survival times for the same age groups were 12.5, 9.6 and 6.4 years, respectively. The difference in survival of these six procedure age categories was significant (p<0.0001; figure 2). Median survival times for AVR alone with no postoperative complication, AVR alone with any complication, AVR+CABG with no complication and AVR+CABG with any complication were 11.07, 7.02, 9.65 and 8.52, respectively. Median survival times stratified by procedural groups, procedure and age groups as well as procedure and EuroSCORE categories are shown in table 3.

**Table 3:** Patient characteristics stratified by procedure (AVR alone, AVR+CABG), age, EuroSCORE and postoperative complication

	Number of patients	Median survival (95% CI)
Entire cohort	1815	10.3 (9.8 to 10.6)
By procedure groups		
AVR alone	967	10.9 (10.5 to 11.8)
AVR+CABG	848	9.6 (8.7 to 10.1)
By procedure and age groups		
AVR alone (65–69)	226	15.1 (13.5, …)
AVR alone (70–79)	563	10.6 (10.1 to 11.6)
AVR alone (≥80)	178	6.3 (5.2 to 8.2)
AVR+CABG (65–69)	174	12.5 (11.2, …)
AVR+CABG (70–79)	485	9.6 (8.6 to 10.1)
AVR+CABG (≥80)	189	6.4 (5.2 to 8.2)
By procedure and EuroSCORE categories		
AVR alone; low EuroSCORE	247	14.5 (13.8, …)
AVR alone; high EuroSCORE	720	9.9 (9.2 to 10.6)
AVR+CABG; low EuroSCORE	182	13.2 (10.7, …)
AVR+CABG; high EuroSCORE	666	8.7 (8 to 9.6)
By procedure groups with or without any postoperative complications*		
AVR alone; no complication	881	11.07 (10.50 to 12.09)
AVR alone; any complication	86	7.02 (5.43 to 10.78)
AVR+CABG; no complication	743	9.65 (8.76 to 10.16)
AVR+CABG; any complication	105	8.52 (6.44, …)

*Postoperative haemofiltration, postoperative stroke and reoperation for bleeding.

AVR, aortic valve replacement; CABG, coronary artery bypass graft; EuroSCORE, European System for Cardiac Operative Risk Evaluation.

Table 4 shows HRs of the predictors of long-term mortality. Age at operation, chronic lung disease, hypertension, diabetes mellitus, extracardiac arteriopathy and preoperative arrhythmia were associated with increased risk of long-term mortality. However, CKD, poor LVF or being a current smoker were each associated with a ≥50% increased risk of long-term mortality.

**Table 4:** Predictors of long-term mortality (Cox regression survival analysis)

Risk factor		HR	p Value
Age at operation		1.1 (1.1 to 1.1)	0.000
Left ventricular function	(Moderately impaired)	1.2 (1.0 to 1.4)	0.119
	(Severely impaired)	1.6 (1.2 to 2.1)	0.002
Lung disease		1.3 (1.1 to 1.6)	0.005
Chronic kidney disease		1.8 (1.2 to 2.8)	0.010
Smoker	(Former)	1.1 (1.0 to 1.3)	0.185
	(Current)	1.5 (1.1 to 2.1)	0.015
Antihypertensive treatment		1.2 (1.0 to 1.4)	0.028
Antidiabetic treatment		1.3 (1.0 to 1.6)	0.056
Arteriopathy (extracardiac)		1.4 (1.1 to, 1.9)	0.016
Arrhythmia (preoperative)		1.4 (1.2 to 1.7)	0.000

‘Gender’ and ‘AVR+CABG versus AVR’ were not significant predictors.

**Figure 3:**
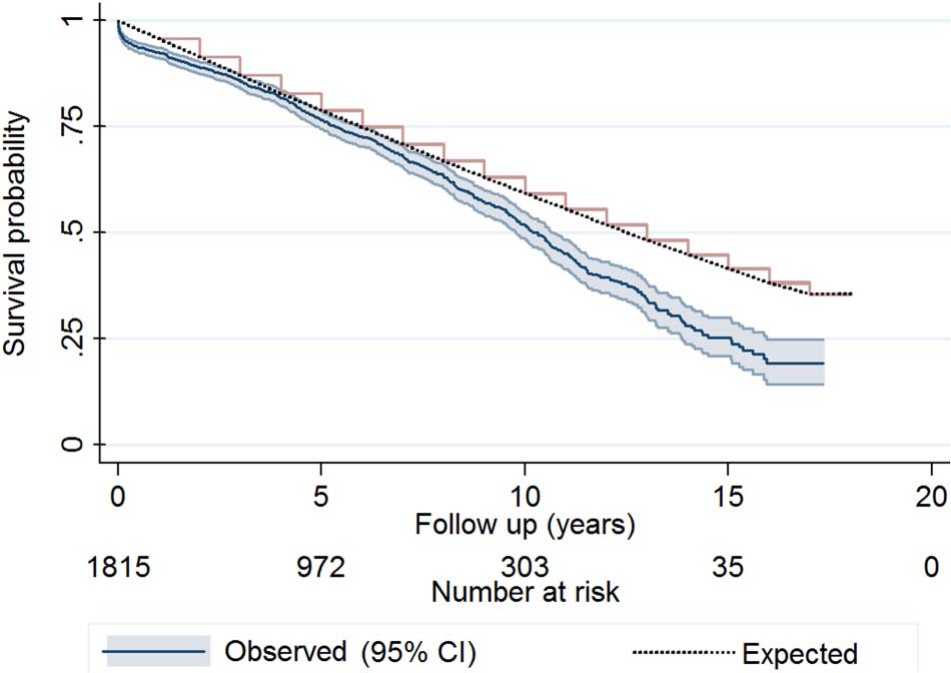
Comparison between Kaplan-Meier survival estimates of Bristol aortic valve surgery patients and the Monte Carlo-based generated Kaplan-Meier curve using the matched ONS population. It appears that for a follow-up period of <8 years, the difference between the survival estimates is not significant (p=0.55) using log-rank test for equality of survivor functions. However, for longer periods of follow-up, the difference becomes increasingly significant with p<0.0001 for the entire follow-up period. ONS, Office for National Statistics.

**Figure 4:**
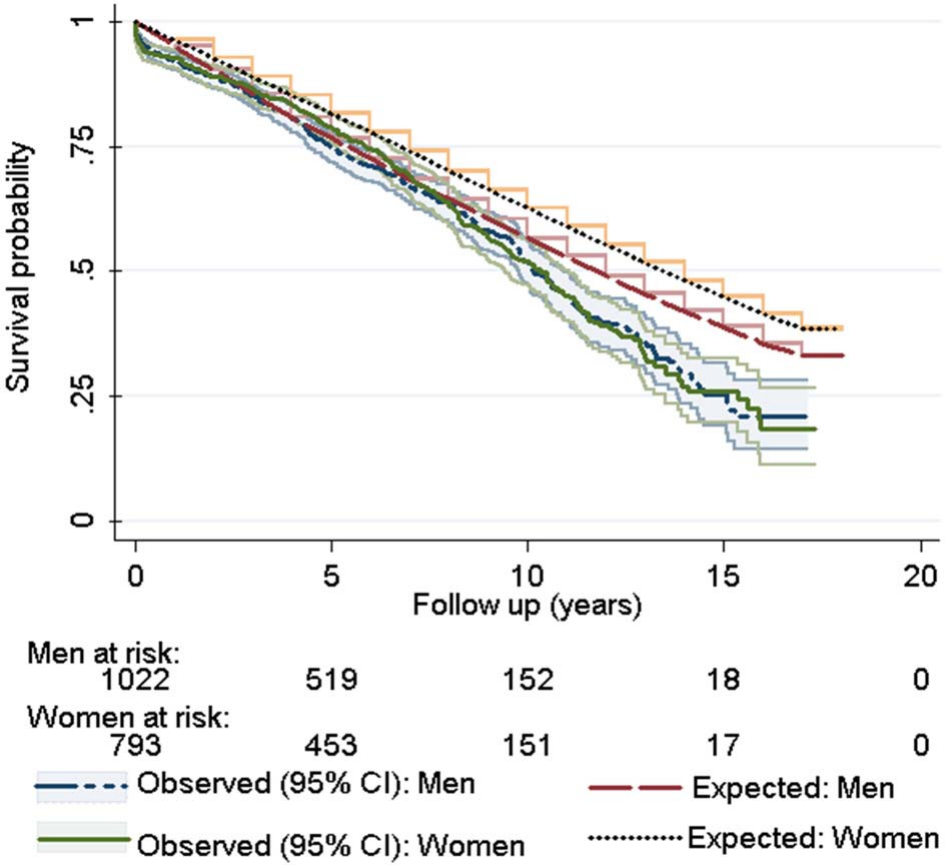
Comparison between Kaplan-Meier survival estimates of Bristol aortic valve surgery patients and the Monte Carlo-based generated Kaplan-Meier curve using the matched ONS population stratified by sex. Whereas the survival probability of women is significantly higher than men in the matched population (p<0.0001), no survival advantage exists for female gender among aortic surgery patients at the age of 65 years and over (p=0.71). ONS, Office for National Statistics.

Comparison of our study cohort with the reference (operation year, age and gender matched) UK population suggested no difference in survival probability up to 8 years (p=0.55). However, for longer periods of follow-up, the difference became increasingly significant (p<0.0001; figure 3). Moreover, the matched UK population shows significantly higher survival probability for women (p<0.0001 for LRT). However, the survival advantage of women no longer existed for aortic valve surgery patients aged 65 years and over (p=0.71; figure 4). We also stratified our analysis by procedural group. In the AVR alone group, survival was not different from the matched general population up to 9 years (p=0.11). However, in the AVR+CABG group, survival matched the general population only up to 7 years (p=0.14). After these points, survival become significantly worse compared to their matched background population in both groups (see online supplementary figure S1).

10.1136/openhrt-2015-000338.supp2 Supplementary figure Comparison between Kaplan-Meier survival estimates of Bristol aortic valve surgery patients and the Monte-Carlo-based generated Kaplan Meier curve using the matched ONS population stratified by operation type (AVR alone vs. AVR + CABG)

## Discussion

### Main findings

Our report provides a detailed contemporary benchmark of long-term survival following AVR among older patients in the UK. From our results, four important findings emerge. First, long-term survival following AVR among older patients is excellent and therefore age should not be a factor in the decision for surgery. Second, baseline CKD, severely impaired LVF and current smoking status indicate particularly poor long-term prognosis after surgical AVR. Third, development of a postoperative complication such as CVA, haemofiltration or re-exploration for bleeding reduced survival compared to patients who did not develop any complication. Fourth, the female survival advantage over men no longer exists in patients undergoing aortic valve surgery.

### Comparison with existing literature

The expected survival of symptomatic patients with severe AS who do not have surgery is 1–4 years.^3^ ^4^ Surgical AVR improves this dismal prognosis and, as we have shown in the present study, restores survival to that expected of an age-matched and gender-matched general population without AS at least up to 8 years of follow-up.

Our findings are consistent with previous reports by high-volume centres undertaking AVR in elderly patients. A report published using the Society of Thoracic Surgeons (STS) Adult Cardiac Surgery Database of over 145 000 patients from over 1000 US centres found a median survival of 13, 9 and 6 years in patients undergoing isolated AVR aged 65–69, 70–79 and ≥80 years of age, respectively.^11^ For patients undergoing AVR+CABG, median survival for the same age groups was 10, 8 and 6 years.^11^ Similarly, baseline CKD, severely impaired LVF and being a current smoker have all been associated with poor long-term survival following surgical AVR.^11^ ^12^

Transcatheter aortic valve implantation (TAVI) has emerged as an alternative to surgical AVR in the treatment of severe AS in inoperable or high-risk surgical candidates. The PARTNER 1B trial has demonstrated the significant short-term and mid-term survival and symptomatic benefit of TAVI compared to medical therapy in inoperable patients with severe AS.^7^ ^13^ In high-risk surgical candidates (defined as an STS risk score of ≥10%), the PARTNER 1A trial demonstrated TAVI to be equivalent to surgical AVR in terms of short-term and mid-term mortality at 2 years.^6^ ^14^ However, TAVI did result in significantly higher rates of stroke, major vascular complications and paravalvular regurgitation compared to surgical AVR. As a result, international valve guidelines now recommend TAVI in patients who have a prohibitive surgical risk and a predicted post-TAVI survival of >12 months or who have a high surgical risk.^15^

Of concern, however, is that TAVI is being extended to low-risk and intermediate-risk surgical patients where no compelling evidence for its efficacy exists. For example, in Germany, the use of TAVI has exploded with 89 TAVIs performed per million population, the highest in Europe.^16^ This is compared to the UK where TAVI use is more regulated and therefore performs <20 implants per million population.^16^ Increasing number of observational studies report the equivalence of TAVI to surgical AVR in terms of in-hospital mortality in low-to-intermediate-risk surgical patients.^17–19^ There are currently three ongoing randomised controlled trials (RCTs; SURTAVI, PARTNER IIA and UK TAVI) that are comparing TAVI and surgical AVR in intermediate-risk patients. The reports of these trials are eagerly awaited; however, the long-term efficacy of TAVI should not merely be measured by equivalence at 1–5 years, but must demonstrate at least equivalence with surgical AVR at 10–15 years follow-up as provided in the current report.

### Strengths and limitations

Our study has three major strengths. First, we used a prospectively maintained clinical database which is managed by several independent database managers who periodically check the accuracy of data entry. Second, we had a low attrition rate with over 98% of patients completing follow-up. Third, our clinical database is linked to the ONS in the UK. This provides accurate mortality data for individual patients as all deaths in the UK are reported to the ONS.

Our report has four limitations. First, we did not have a comparison group which would ideally be patients who have undergone TAVI with long-term follow-up data. However, at present, such data are not available. Nevertheless, we compared our cohort survival with operation year-matched, age-matched and gender-matched general population. Second, we did not report the incidence of structural valve deterioration and the need for reoperation as these data are not readily available through our database. However, the deterioration of survival after 8 years of follow-up may be in part indicative of structural valve deterioration and the need for reoperation. Third, we did not conduct long-term functional assessment of patients which would be important for benchmarking. Last, we did not have cause of late mortality.

Our study highlights the need for national registries of surgical AVR and TAVI that provide detailed information on patient and procedural characteristics. Such registries must be linked to national mortality data so that long-term outcomes in patients with AS can be accurately reported.

## Conclusion

Long-term survival following surgical AVR in patients over 65 years of age is excellent and is comparable to the matched general population especially up to 8 years. Baseline CKD, severely impaired LVF and current smoking status are associated with a particularly poor prognosis.
