# Supplementary material for: Long-term survival after surgical aortic valve replacement among patients over 65 years of age
Source: Open Heart. 2016 Mar 25;3(1):e000338. doi: 10.1136/openhrt-2015-000338 (PMC4809186; doi:10.1136/openhrt-2015-000338)
Supplement: Supplementary table — Univariate Logistic Regression [file openhrt-2015-000338supp_table.pdf]

**Age**				
	70-79	1.8	(1.4, 2.3)	< 0.0001
	>80	2.7	(1.9, 3.6)	< 0.0001
**Left Ventricular Function**				
	Moderate	1.7	(0.9, 3.0)	0.095
	Poor	2.5	(1.1, 5.4)	0.023
**Chronic Kidney Disease**		3.1	(1.1, 9.1)	0.035
**Chronic Lung Disease**		1.8	(1.0, 3.2)	0.058
**AVR + CABG**		1.8	(1.1, 2.9)	0.024
**Diabetes**		1.2	(0.6, 2.0)	0.546
**Anti-Hypertension treatment**		1.3	(0.8, 2.2)	0.354
**Arteiopathy**		2.0	(0.9, 4.4)	0.066
**Arrythmia**		1.9	(1.1, 3.3)	0.032
**Smoking**				
	Former	1.4	(0.9, 2.4)	0.154
	Current	0.7	(0.1, 2.8)	0.564
**Euroscore**		1.4	(1.2, 1.5)	< 0.0001

Supplementary Table 1: Univariate Logistic Regression
