# Supplementary material for: Long-term survival after surgical aortic valve replacement among patients over 65 years of age
Source: Open Heart. 2016 Mar 25;3(1):e000338. doi: 10.1136/openhrt-2015-000338 (PMC4809186; doi:10.1136/openhrt-2015-000338)
Supplement: Supplementary figure — Comparison between Kaplan-Meier survival estimates of Bristol aortic valve surgery patients and the Monte-Carlo-based generated Kaplan Meier curve using the matched ONS population stratified by operation type (AVR alone vs. AVR + CABG) [file openhrt-2015-000338supp_figure.pdf]

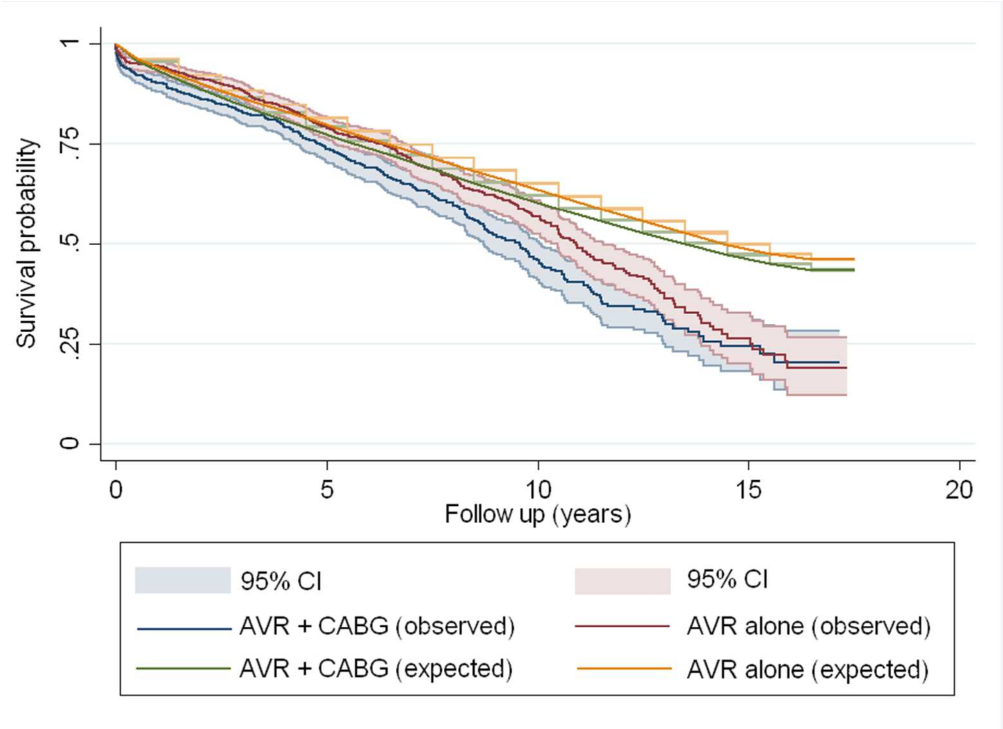

Comparison between Kaplan-Meier survival estimates of Bristol aortic valve surgery patients and the Monte-Carlo-based generated Kaplan Meier curve using the matched ONS population stratified by operation type (AVR alone vs. AVR + CABG)
